# The diagnostic accuracy of ultrasonography in pediatric intussusception and its role in predicting the need for surgery

**DOI:** 10.3389/fped.2026.1940450

**Published:** 2026-10-07

**Authors:** Murat Solmaz, Osman Gultekin, Ozhan Orhan, Devran Demir

**Affiliations:** 1 Department of Pediatrics, Batman Iluh State Hospital, Batman, Turkiye; 2 Department of Radiology, Batman Training and Research Hospital, Batman, Türkiye; 3 Department of Pediatrics, Mardin Artuklu University, Mardin, Turkiye; 4 Department of Pediatrics, Devran Demir Clinics, İzmir, Türkiye

**Keywords:** diagnostic accuracy, intussusception, need for surgery, pediatric, ultrasound

## Abstract

**Objective:**

Pediatric intussusception is a common gastrointestinal emergency requiring early diagnosis and appropriate treatment. Although ultrasonography (USG) is considered the first-line imaging modality for diagnosis, its potential value in identifying patients who may ultimately require surgical treatment remains uncertain. This study aimed to evaluate the diagnostic accuracy of ultrasonography in children with suspected intussusception and investigate associations of ultrasonographic findings and laboratory parameters with surgical management.

**Methods:**

In this retrospective, single-center study, 113 patients with suspected intussusception who underwent abdominal ultrasound at the time of presentation between January and December 2025 were evaluated. The patients’ demographic characteristics, clinical findings, laboratory parameters, ultrasound findings, treatment methods, and clinical outcomes were analyzed. Diagnostic performance was assessed using a composite clinical reference standard based on operative confirmation, successful non-invasive reduction with clinical improvement, or exclusion of intussusception during documented clinical follow-up. Factors associated with surgical management were evaluated using comparative analyses and ROC curves.

**Results:**

The mean age of the 113 patients was 2.3 ± 1.8 years, and 68.1% were male. Intussusception was detected on ultrasonography in 52 patients (46.0%). Non-invasive reduction was initially attempted in 47 of 52 patients; 42 were successfully reduced on the first attempt, while 5 proceeded to surgery after failed reduction. Five additional patients underwent primary surgery, resulting in 10 patients (19.2%) receiving surgical treatment. In patients who underwent surgery, intussuscepted segment length, free intraperitoneal fluid, white blood cell count, C-reactive protein level, and neutrophil-to-lymphocyte ratio were significantly higher (*p* < 0.05 for all comparisons). In ROC analysis, cutoff values of 39.5 mm for intussuscepted segment length and 4.8 mg/dL for C-reactive protein demonstrated good discriminatory performance between final treatment groups.

**Conclusion:**

Ultrasonography demonstrated high diagnostic accuracy for pediatric intussusception in this retrospective cohort. Longer intussuscepted segment length, free intraperitoneal fluid, and higher inflammatory markers were associated with surgical management. These findings may be useful for future risk-stratification approaches but should not be considered stand-alone indications for surgery. Prospective multicenter studies are needed to validate their clinical utility.

## 1 Introduction

Intussusception is one of the most common causes of intestinal obstruction in childhood and is a gastrointestinal emergency that can lead to significant morbidity and mortality, particularly in children between 6 months and 3 years of age. If the condition is not recognized early, life-threatening complications such as intestinal edema, ischemia, necrosis, perforation, and sepsis may develop. Therefore, early diagnosis and an appropriate treatment approach are critical for improving clinical outcomes (1, 2).

In diagnosis, ultrasound (US) is the imaging method of first choice for suspected pediatric intussusception because it is radiation-free, readily available, allows for real-time assessment, and has high diagnostic accuracy (3, 4). In particular, the “target sign” and “pseudokidney” appearance are considered characteristic ultrasonographic findings for intussusception; in previous studies, the sensitivity and specificity of USG have been reported to range from 92%–100% and 88%–100%, respectively (5–7). In addition, ultrasound offers significant advantages not only in diagnosis but also in guiding reduction procedures and monitoring the course of treatment.

However, much of the existing literature has focused on the diagnostic accuracy of ultrasonography, and the prognostic value of ultrasonographic findings in predicting the need for surgical treatment has not been sufficiently investigated (8, 9). In particular, there are limited studies evaluating the relationship between ultrasonographic features—such as the length of the invaginated segment, free intraperitoneal fluid, intestinal wall thickness, and the pathological leading edge—and clinical outcomes. Furthermore, there is no definitive consensus regarding the potential benefits that evaluating inflammatory markers in conjunction with ultrasound findings could provide for patient management (10–12). Therefore, there is a need to identify reliable markers that will enable early risk stratification of patients.

The aim of this study is to evaluate the diagnostic accuracy of ultrasound in children presenting with suspected pediatric intussusception, to investigate the value of ultrasound findings in predicting the need for surgical treatment, and to demonstrate the contribution of the combined evaluation of laboratory markers and ultrasound findings to the clinical decision-making process. Thus, our study aims to contribute to the existing literature regarding the use of ultrasound in the management of pediatric intussusception, not only for diagnostic purposes but also for prognostic purposes. A secondary objective was to explore the discriminatory performance of these variables as candidate factors for future risk-stratification studies, rather than to establish a definitive clinical prediction rule.

## 2 Materials and methods

### 2.1 Study design and patient population

This retrospective, observational, single-center study was conducted among pediatric patients who presented to the Batman Training and Research Hospital Emergency Department with suspected intussusception between January 2025 and December 2025. A total of 113 pediatric patients who underwent abdominal ultrasound (USG) at the time of presentation and for whom complete clinical, laboratory, and imaging data were available were included in the study. Patients with a history of intussusception, chronic inflammatory bowel disease such as Crohn's disease or ulcerative colitis, acute abdominal conditions resulting from trauma, ultrasound examinations that could not be technically evaluated, or incomplete clinical data were excluded from the study. Patient data were retrospectively obtained from the electronic hospital information management system and the digital radiology archive. The study was prepared in accordance with the STROBE (Strengthening the Reporting of Observational Studies in Epidemiology) guidelines for reporting observational studies.

### 2.2 Data collection

Patients’ demographic characteristics (age, sex, and time of presentation), clinical findings (abdominal pain, vomiting, bloody stools, restlessness, lethargy, fever, duration of symptoms, and physical examination findings), laboratory results, and ultrasound findings were obtained from the electronic medical record system. Laboratory evaluations included a complete blood count, C-reactive protein (CRP), albumin, electrolytes, and blood gas parameters. Additionally, the neutrophil-to-lymphocyte ratio (NLR), platelet-to-lymphocyte ratio (PLR), lymphocyte-to-CRP ratio (LCR), and CRP-to-albumin ratio (CAR) were calculated to assess the systemic inflammatory response.

Ultrasound reports were reviewed for the presence and type of intussusception, the length of the intussuscepted segment, the thickness of the intussuscepted head and body, the target sign, the pseudokidney sign, free intraperitoneal fluid, bowel wall thickening, mesenteric lymphadenopathy, and the presence of a pathological lead point. Clinical outcomes included the initial treatment strategy, success or failure of non-invasive reduction, final surgical management, length of hospital stay, 30-day recurrence, and mortality. Thirty-day recurrence was assessed retrospectively from follow-up information documented in the institutional electronic medical record. A standardized prospective or telephone-based follow-up protocol was not used, and a formal follow-up completion rate was not available; therefore, recurrence events managed exclusively at another institution may not have been captured.

### 2.3 Ultrasonographic evaluation

Abdominal ultrasonographic examinations were performed in our hospital's radiology department as part of routine clinical practice using a high-frequency linear probe (8–12 MHz) and, when necessary, a low-frequency convex probe (3–5 MHz). The diagnosis of intussusception was established by identifying the characteristic “target sign” appearance in the transverse plane or the “pseudokidney sign” appearance in the longitudinal plane. In addition, the length of the intussuscepted segment, the thickness of the intussuscepted head and body, the presence of free fluid, bowel wall thickening, mesenteric lymph nodes, and the pathological leading edge were evaluated in detail.

### 2.4 Treatment approach and reference standard

In hemodynamically stable patients without signs of perforation or generalized peritonitis, hydrostatic or pneumatic reduction was performed as the initial treatment when clinically appropriate. Surgical treatment was performed after failed non-invasive reduction or when a primary indication for surgery was present. The first ultrasonographic examination at presentation was considered the index test. The final diagnosis was assessed using a composite clinical reference standard: intussusception was considered confirmed when documented during surgery or when successful non-invasive reduction was followed by clinical improvement; exclusion was based on the subsequent documented clinical course when intussusception-specific treatment was not required. For patients with negative ultrasonographic findings, the available emergency department or inpatient course and subsequent institutional records were reviewed, when available, for persistent or recurrent symptoms and a later diagnosis of intussusception. Because this was a retrospective study, a uniform prespecified follow-up interval and an independent reference test were not available for every ultrasound-negative patient. In addition, ultrasonographic findings influenced subsequent management. Accordingly, partial verification and incorporation bias cannot be excluded.

### 2.5 Treatment outcome definitions and follow-up

Initial treatment strategy and final treatment outcome were analyzed as distinct clinical concepts. Among the 52 patients in whom intussusception was detected on ultrasonography, non-invasive reduction was initially attempted in 47 patients and primary surgery was performed in 5. Of the 47 attempted reductions, 42 were successful on the first attempt and 5 failed and proceeded to surgery. Thus, the final successful non-invasive treatment group comprised 42 patients, whereas the final surgical group comprised 10 patients (5 failed reductions plus 5 primary surgeries). These final outcome groups were used for the comparative analyses and for the exploratory ROC analyses. Thirty-day recurrence was evaluated from available institutional records as described above.

### 2.6 Statistical analysis

Statistical analyses were performed using IBM SPSS Statistics for Windows, Version 25.0 (IBM Corp., Armonk, NY, USA). The normality of continuous variables was assessed using the Kolmogorov–Smirnov test. Normally distributed data were presented as mean ± standard deviation, whereas non-normally distributed data were presented as median (interquartile range). Categorical variables were expressed as counts and percentages. For between-group comparisons, the chi-square test or Fisher's exact test was used for categorical variables, and the independent-samples t-test or Mann–Whitney U test was used for continuous variables, as appropriate.

Associations with final surgical management were evaluated using univariable group comparisons. ROC curve analyses were exploratory, and optimal cutoff values were determined using the Youden index. Because only 10 final surgical outcomes occurred, no multivariable prediction model was fitted. All tests were two-tailed, and *p* < 0.05 was considered statistically significant.

## 3 Results

A total of 113 pediatric patients were included in the study. Patient flow, ultrasonographic findings, and treatment outcomes are summarized in Figure 1. The mean age of the patients was 2.3 ± 1.8 years, and 68.1% of the cases were male. The most common symptoms at presentation were abdominal pain (90.3%) and vomiting (62.8%). Abdominal tenderness (71.7%) was the most frequent physical examination finding. Presentations were more frequent during spring and summer, which together accounted for 59.3% of cases. The demographic and clinical characteristics of the study population are presented in Table 1. Laboratory parameters at presentation, grouped by ultrasonographic findings, are presented in Table 2. The sonographic characteristics of the 52 patients with ultrasonographically detected intussusception are summarized in Table 3. The diagnostic performance measures of ultrasonography are presented in Table 4.

**Figure 1:**
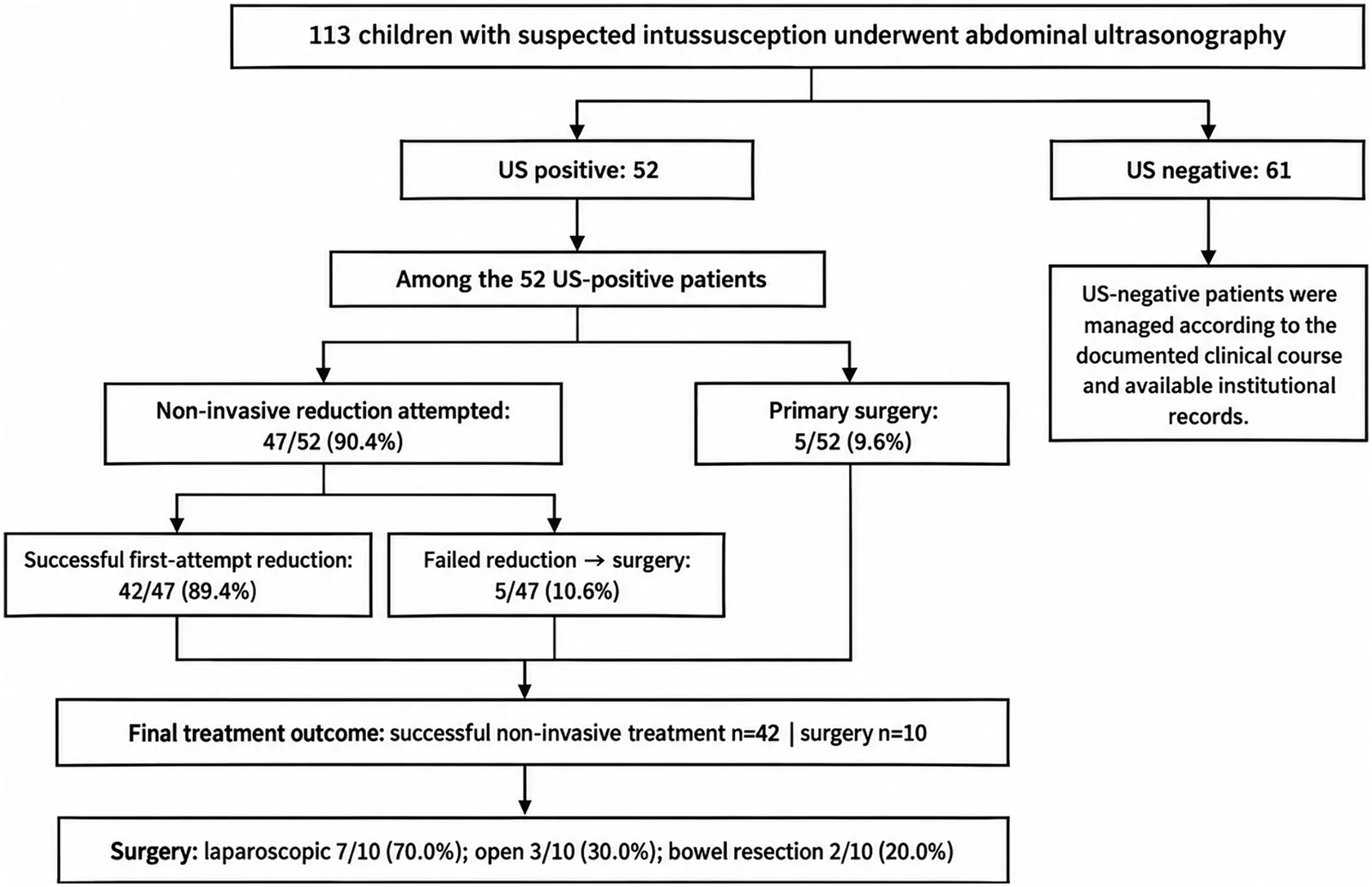
Patient flow and treatment outcomes.

**Table 1:** Demographic and clinical characteristics of patients.

Variable	Value
Age (years), mean ± SD (median)	2,3 ± 1,8 (1,8)
0–6 months	6 (%5,3)
6–12 months	28 (%24,8)
1–3 years	51 (%45,1)
3–5 years	19 (%16,8)
>5 years	9 (%8,0)
Gender	
Male	77 (%68,1)
Female	36 (%31,9)
Symptoms	
Abdominal pain	102 (%90,3)
Vomiting	71 (%62,8)
Bloody stool	38 (%33,6)
Restlessness/agitation	64 (%56,6)
Lethargy	22 (%19,5)
Fever (>38 °C)	29 (%25,7)
Duration of symptoms (hours), median (distribution)	18 (2–96)
Physical examination findings	
Abdominal tenderness	81 (%71,7)
Abdominal distension	26 (%23,0)
Tachycardia	17 (%15,0)
Signs of peritonitis	9 (%8,0)
Season of presentation	
Spring	35 (%31,0)
Summer	32 (%28,3)
Fall	24 (%21,2)
Winter	22 (%19,5)

Leukocytosis was detected in 47.8% of patients at presentation. Patients in the intussusception group had significantly higher WBC count, neutrophil count, CRP level, and NLR and lower albumin levels than those in the comparison group (*p* < 0.05 for all comparisons).

**Table 2:** Laboratory parameters at the time of application.

Parameter	All patients (*n* = 113)	Intussusception (+) (*n* = 52)	Intussusception (-) (*n* = 61)	*p*-value
Complete Blood Count				
WBC (x10^3^/*μ*L)	12,4 ± 4,2	14,1 ± 4,6	11,0 ± 3,2	<0,001
Neutrophils (x10^3^/μL)	7,8 ± 3,2	9,2 ± 3,5	6,6 ± 2,5	<0,001
Lymphocytes (x10^3^/μL)	3,9 ± 1,7	3,7 ± 1,6	4,1 ± 1,8	0,212
Platelets (x10^3^/μL)	312 ± 98	321 ± 102	304 ± 94	0,367
Inflammatory markers				
CRP (mg/dL)	3,2 ± 2,8	4,5 ± 3,1	2,1 ± 2,0	<0,001
Albumin (g/dL)	4,1 ± 0,4	4,0 ± 0,4	4,2 ± 0,3	0,018
NLR	2,5 ± 1,2	3,0 ± 1,3	2,1 ± 1,0	<0,001

Data are presented as mean ± SD.

WBC, white blood cells; CRP, C-reactive protein; NLR, neutrophil-to-lymphocyte ratio.

Intussusception was detected in 52 patients (46.0%) on abdominal ultrasonography. Among these patients, the most frequently recorded sonographic findings were the target sign (90.4%) and pseudokidney sign (71.2%). Most cases were ileocolic (67.3%).

**Table 3:** Detailed findings of patients with intussusception detected by ultrasound (*n* = 52).

Findings	n (%)/Value
Type of intussusception	
Ileocolic	35 (%67,3)
Small intestine	11 (%21,2)
Ileo-ileocolic	6 (%11,5)
Ultrasound appearance	
Target sign	47 (%90,4)
Pseudokidney	37 (%71,2)
Free fluid	23 (%44,2)
Intestinal wall thickening	19 (%36,5)
Lymphadenopathy	14 (%26,9)
Other	6 (%11,5)
Intussusception parameters (mm), mean ± SD	
Length of intussuscepted segment	35,4 ± 12,3
Thickness of intussusception head	14,2 ± 4,1
Thickness of intussusception body	9,8 ± 3,2

**Table 4:** Diagnostic performance measures.

Measure	Value (%, n/N)	95% Confidence Interval
Sensitivity	94,2 (49/52)	84,1–98,4
Specificity	91,8 (56/61)	82,0–96,7
Positive predictive value (PPV)	89,1 (49/55)	78,8–94,8
Negative predictive value (NPV)	96,6 (56/58)	86,8–98,9
Accuracy	92,9 (105/113)	86,6–96,6

Among the 52 patients with ultrasonographically detected intussusception, non-invasive reduction was used as the initial treatment in 47/52 (90.4%). Hydrostatic reduction was performed in 29/47 (61.7%) and pneumatic reduction in 18/47 (38.3%). First-attempt reduction was successful in 42/47 patients (89.4%); the remaining 5/47 (10.6%) proceeded to surgery after failed reduction. Five additional patients underwent primary surgery (5/52, 9.6%), resulting in 10/52 patients (19.2%) receiving final surgical management. Of the 10 surgical procedures, 7/10 (70.0%) were laparoscopic and 3/10 (30.0%) were open; bowel resection was performed in 2/10 (20.0%). Treatment methods and outcomes are summarized in Table 5. Mean hospital stay was longer in the surgical group than in the successful non-invasive treatment group (6.8 ± 2.5 vs. 2.4 ± 1.1 days, *p* < 0.001). Within 30 days, recurrence was documented in 3/42 patients (7.1%) after successful non-invasive reduction and in 0/10 patients after surgery. Because follow-up was retrospective and not standardized, these recurrence figures represent documented events in the available institutional records. No mortality was observed during the study period.

**Table 5:** Treatment methods and outcomes in patients with ultrasonographically detected intussusception (*n* = 52).

Treatment method or outcome	Value
Non-invasive reduction (initial treatment)	47/52 (90.4%)
– Hydrostatic reduction	29/47 (61.7%)
– Pneumatic reduction	18/47 (38.3%)
Successful non-invasive reduction on the first attempt	42/47 (89.4%)
Failed non-invasive reduction → surgery	5/47 (10.6%)
Primary surgery	5/52 (9.6%)
Final surgical management	10/52 (19.2%)
– Laparoscopic reduction	7/10 (70.0%)
– Open reduction	3/10 (30.0%)
– Bowel resection	2/10 (20.0%)
Length of hospital stay (days), mean ± SD	
Non-invasive reduction	2,4 ± 1,1
Surgery	6,8 ± 2,5
Recurrence within 30 days	
Successful non-invasive treatment	3/42 (7.1%)
Surgery	0/10 (0.0%)

For analyses of final treatment outcome, the surgical group (*n* = 10; 5 failed reductions and 5 primary surgeries) was compared with the successful non-invasive treatment group (*n* = 42), as presented in Table 6. The surgical group had a higher mean age and a longer duration of symptoms. Abdominal pain, vomiting, and bloody stools were also more frequent in the surgical group in the univariable comparisons (*p* < 0.05 for all comparisons).

**Table 6:** Comparison of final treatment outcome groups among patients with ultrasonographically detected intussusception.

Variable	Surgery (*n* = 10)	Successful non-invasive treatment (*n* = 42)	*p*-value
Demographic			
Age (years)	3,9 ± 1,6	1,8 ± 1,4	<0,001
Male gender	8 (%80)	29 (%69)	0,493
Clinical			
Duration of symptoms (hours)	36,5 ± 12,8	18,2 ± 8,4	<0,001
Abdominal pain	10 (%100)	37 (%88,1)	0,025
Vomiting	9 (%90)	24 (%57,1)	0,048
Bloody stool	7 (%70)	11 (%26,2)	0,011
Ultrasound findings			
Length of invaginated segment (mm)	45,2 ± 10,1	30,5 ± 9,8	<0,001
Thickness of invaginated head (mm)	16,5 ± 4,2	13,2 ± 3,8	0,024
Free fluid	7 (%70)	12 (%28,6)	0,017
Laboratory			
WBC (x10^3^/μL)	16,8 ± 4,1	12,5 ± 3,8	0,003
Neutrophils (x10^3^/μL)	11,2 ± 3,4	7,5 ± 2,9	0,002
CRP (mg/dL)	7,2 ± 3,5	3,2 ± 2,4	<0,001
Albumin (g/dL)	3,8 ± 0,4	4,1 ± 0,3	0,032
NLR	3,8 ± 1,4	2,4 ± 1,0	0,001

Data are presented as mean ± SD or n (%).

In the ultrasonographic evaluation, greater intussuscepted segment length, greater head thickness, and the presence of free intraperitoneal fluid were associated with final surgical management. Higher WBC count, neutrophil count, CRP level, and NLR and lower albumin level were also associated with the surgical group in univariable analyses.

In exploratory ROC analyses, the optimal cutoff values were 39.5 mm for intussuscepted segment length (AUC=0.89; sensitivity 90%, specificity 81%) and 4.8 mg/dL for CRP (AUC = 0.85; sensitivity 85%, specificity 78%). Because these thresholds were derived from the same retrospective cohort and only 10 surgical outcomes occurred, they should be considered exploratory rather than validated clinical decision thresholds.

## 4 Discussion

This study found that ultrasonography had high diagnostic performance in children presenting with suspected pediatric intussusception when evaluated using the composite clinical reference standard applied in routine practice. In addition, several sonographic and laboratory findings were associated with final surgical management. In particular, greater intussuscepted segment length and free intraperitoneal fluid, together with higher CRP and NLR values, were more frequent in the surgical group. Given the retrospective single-center design and the small number of surgical outcomes, these findings should be interpreted as associations and hypothesis-generating observations rather than validated predictors of the need for surgery (13–17).

Our results also support the use of ultrasound as the first-line imaging method for pediatric intussusception.

A notable finding was the association between intussuscepted segment length and final surgical management. Segment length was greater in the surgical group, and a threshold of 39.5 mm showed good discrimination in exploratory ROC analysis. From a pathophysiological perspective, a longer intussuscepted segment may be associated with greater mesenteric compression, impaired venous return, bowel wall edema, and ischemic change, which may reduce the likelihood of successful non-invasive reduction. Previous studies have similarly reported associations between longer intussusception segments, failed reduction, and operative intervention (18, 19). However, the cutoff identified in our cohort is internally derived and should not be used as a stand-alone surgical threshold without external validation.

Free intraperitoneal fluid was also more frequent in patients who underwent surgery. This finding may reflect more advanced bowel wall edema, venous congestion, or inflammatory change. Although free fluid alone is not an indication for surgery, its presence may identify a subgroup with more severe disease when considered together with the clinical examination and other imaging findings (18–20).

CRP and NLR levels were significantly higher in the surgical group. Ischemia and inflammation in the intussuscepted bowel can trigger a systemic inflammatory response, providing a plausible biological explanation for these associations. In the present study, CRP showed good exploratory discrimination, whereas NLR was higher in patients receiving surgical management. These markers may be useful candidate variables for future risk-stratification models, but the current data do not establish them as independent predictors or treatment-directing biomarkers (19, 21).

Taken together, intussuscepted segment length, free intraperitoneal fluid, and inflammatory markers may provide complementary information about disease severity. Their combined assessment could be investigated in future prospective risk-stratification models; however, the present study does not support using these variables to select patients for surgery, to bypass an otherwise appropriate non-invasive reduction attempt, or to replace established clinical and surgical criteria. Clinical management should continue to integrate hemodynamic status, signs of perforation or peritonitis, imaging findings, reduction response, and multidisciplinary judgment (13, 17, 22).

To the best of our knowledge, this study is among the limited reports from our country evaluating diagnostic performance together with sonographic and laboratory features associated with treatment outcome in pediatric intussusception. Evaluation in different patient populations is important because local referral patterns, timing of presentation, operator experience, and treatment protocols may affect observed associations (13, 23). The present findings therefore provide hypothesis-generating data that may inform the design of larger prospective studies.

This study has several limitations. First, the retrospective, single-center design limits generalizability and is susceptible to missing or non-standardized clinical information. Second, only 10 patients underwent final surgical management, limiting statistical power and precluding reliable multivariable modeling. Third, the diagnostic reference standard was composite and was not independent of the index ultrasonography in all patients. Ultrasound findings influenced subsequent reduction or surgical decisions, and ultrasound-negative patients did not undergo a uniform independent verification procedure or a standardized prespecified follow-up interval; therefore, partial verification and incorporation bias are possible. In addition, the retained retrospective analytic data did not permit reliable reconstruction of a complete patient-level cross-tabulation of index-test and reference-standard status without *post hoc* assumptions, so the original summary diagnostic performance estimates were retained. Fourth, 30-day recurrence was assessed from available institutional records rather than a standardized prospective or telephone-based follow-up protocol, and events managed exclusively elsewhere may have been missed. Finally, the ROC thresholds were derived and evaluated in the same cohort and should be considered exploratory. These limitations underscore the need for prospective multicenter validation.

In summary, ultrasonography showed high diagnostic performance in this retrospective cohort. Greater intussuscepted segment length, free intraperitoneal fluid, and higher inflammatory markers were associated with final surgical management. These findings are hypothesis generating and may support the development of future risk-stratification approaches, but they should not be interpreted as validated predictors or as stand-alone criteria for surgery. Prospective multicenter studies with standardized diagnostic verification and follow-up are required.

## 5 Conclusion

Ultrasonography showed high diagnostic performance for pediatric intussusception in this retrospective single-center cohort. Longer intussuscepted segment length, free intraperitoneal fluid, and higher inflammatory markers were associated with final surgical management. These findings may inform future risk-stratification research but should not be used as stand-alone indications for surgery. Prospective multicenter studies with standardized diagnostic verification and external validation are required before these associations can be incorporated into clinical decision tools.

## Data Availability

The raw data supporting the conclusions of this article will be made available by the authors, without undue reservation.
